# Chylous Ascites Developing after Open Thoracoabdominal Aortic Aneurysm Repair in a Patient with Marfan Syndrome

**DOI:** 10.21470/1678-9741-2019-0019

**Published:** 2020

**Authors:** Hakan Kara

**Affiliations:** 1 Department of Cardiovascular Surgery, Giresun Ada Hospital, Giresun, Turkey.

**Keywords:** Chylous Ascites, Aortic Aneurysm, Thoracic, Thoracic Duct, Marfan Syndrome, Abdominal Pain, Elective Surgical Procedures, Lymphoma, Tomography

## Abstract

Chylous ascites is the pathologic accumulation of chylous fluid in the peritoneal cavity, caused by lymphomas, metastatic malignancies, and abdominal surgeries, rarely due to surgical trauma of the cisterna chyli or its major branches. A 24-year-old man with history of Marfan syndrome presented to our hospital with abdominal distention, abdominal pain, fluid in the incision region, and weakness. He had underwent an elective open aneurysm repair surgery nine days before for thoracoabdominal aortic aneurysm. Computed tomography revealed massive fluid collection in the abdominal cavity, which was drained surgically. He was diagnosed with chylous ascites and was discharged after conservative treatment.

**Table:** 

Abbreviations, acronyms & symbols	
AAA	= Abdominal aortic aneurysm
ALT	= Alanine transaminase
AST	= Aspartate aminotransferase
CRP	= C-reactive protein
CT	= Computed tomography
LDH	= Lactate dehydrogenase

## INTRODUCTION

Chylous ascites developing after open surgical repair of abdominal aortic aneurysm (AAA) is a rare and serious condition. It is defined as the pathologic accumulation of chylous fluid in the peritoneal cavity^[1]^. The accumulation of chyle within the peritoneal cavity may cause severe discomfort to the patient, resulting in complication during the postoperative course^[2]^. This disease should, therefore, be taken into consideration in patients who show persistent abdominal distention after open AAA and retroperitoneal surgery^[3]^. We have presented here a rare case of chylous ascites after open thoracoabdominal aortic aneurysm repair surgery.

## CASE REPORT

**Fig. 1:**
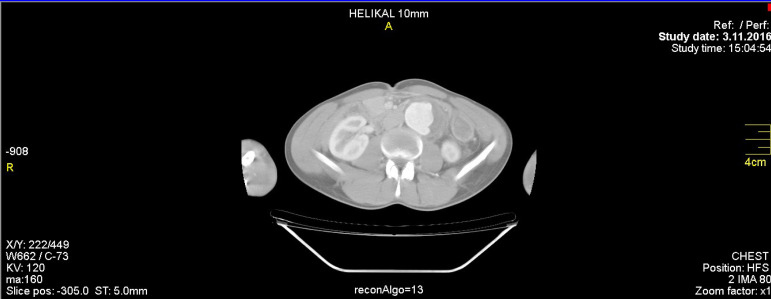
Preoperative thoracoabdominal computed tomography. Thoracoabdominal aortic aneurysm.

**Fig. 2:**
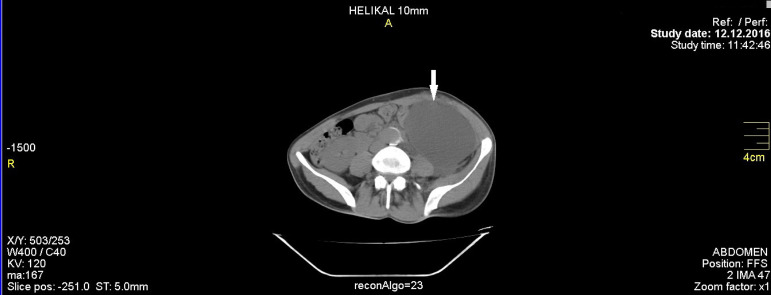
Postoperative thoracoabdominal computed tomography. Massive chylous fluid collection in the abdominal cavity.

**Fig. 3:**
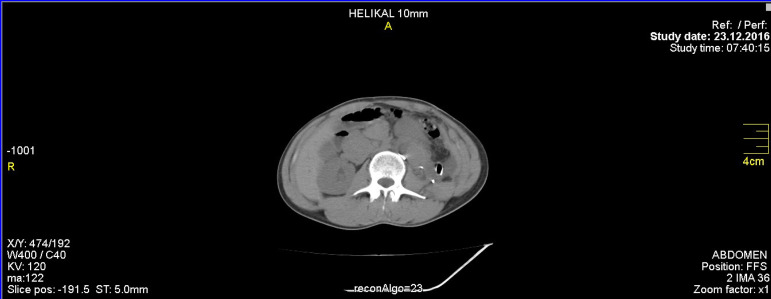
Thoracoabdominal computed tomography before discharge.

A 24-year-old man presented to our hospital with abdominal distention, abdominal pain, fluid in the incision region, and weakness. This patient, with Marfan syndrome history and whose complaints increased during the last three days, had undergone an open aneurysm repair surgery in a different institution nine days before his presentation, after being diagnosed with thoracoabdominal aortic aneurysm (Figure1). His medical history revealed that he also had undergone elective ascending aorta replacement and mechanical aortic valve replacement surgery 10 years ago. Physical examination revealed abdominal distention with hyperactive bowel sounds. There was tenderness in the abdomen on palpation and white-colored fluid in the incision region. His body temperature was 36.8ºC, blood pressure was 100/60 mmHg, and heart rate was 90 bpm. His electrocardiogram was normal, except for a few premature extra atrial systoles. Furthermore, his laboratory tests revealed the following: white blood cell 17,820/µL, hemoglobin 11.9 g/dL, hematocrit 28%, platelet 642,000/µL, glucose 88 mg/dL, sodium 136 mEq/L, potassium 4.45 mEq/L, creatinine 0.65 mg/dL, aspartate aminotransferase (AST) 40 IU/L, alanine transaminase (ALT) 44 IU/L, C-reactive protein (CRP) 6.35 mg/dL, and an international normalized ratio of 2.04. No abnormal findings were detected on his chest X-ray. Computed tomography (CT) scan of his abdomen revealed massive fluid collection (measuring 21 × 10 × 8 cm) in the abdominal cavity of an unknown origin (Figure 2). Culture antibiogram was received from the flow incision region. The patient had high fever twice (38.1 and 38.4ºC) before chylous ascites was drained. Ampicilline 1 g + sulbactam 0.5 g were administered to the patient intravenously, four times daily. Under general anesthesia, the abdomen was opened by removing some of the sutures on the skin that remained from the open thoracoabdominal aortic repair surgery performed nine days before. Up to 2400 ml of chylous ascites was drained, and one drainage tube was placed in the retroperitoneum. Analysis of the fluid revealed a glucose level of 101 mg/dL, lactate dehydrogenase (LDH) level of 97 IU/L, total protein level of 6.3 g/dL, cholesterol level of 58 mg/dL, and triglyceride level of 885 mg/dL. There was no reproduction in the culture of the patient, who did not have malignant cells in the peritoneal fluid cytology. The patient’s chylous ascites diagnosis was thereby confirmed. His oral intake was discontinued, and the total parenteral nutrition was initiated. In the first three days of his daily follow-ups, chylous fluid drainage of 400-700 mL was recorded; however, on the 4^th^ day, somatostatin was added because of insufficient chylous flow reduction. A loading dose of somatostatin of 3.5 mcg/kg dissolved in 2 ml of 0.9 % sodium chloride was administered intravenously, infused within 10 minutes. The treatment was continued with an intravenous infusion rate of 3.5 mcg/kg/h for four days. On the 11^th^ day of admission, the effusion amount was <50 mL and the drainage tube was removed from the abdomen. On the 8^th^ day of hospitalization, oral intake was started when somatostatin treatment was completed. Patient’s CRP values until discharge were as follows: 1^st^ day, 6.31 mg/dL; 3^rd^ day, 4.21 mg/dL; 5^th^ day, 2.56 mg/dL; 8^th^ day, 1.25 mg/dL; 12^th^ day, 0.61 mg/dL; and 17^th^ day, 0.65 mg/dL. The white blood cell count was as follows: 1^st^ day, 10240/µL; 3^rd^ day, 14740/µL; 5^th^ day, 7360/µL; 8^th^ day, 4500/µL; 12^th^ day, 6010/µL; and 17^th^ day, 8330/µL. The patient was discharged on the 17^th^ postoperative day. He was completely free from symptoms for one month, his ultrasound scan and CT scan revealed no sign of ascites (Figure 3).

## DISCUSSION

Chylous ascites is the accumulation of chyle in the peritoneal cavity and its major causes are lymphomas, metastatic malignancies, and abdominal surgeries. This disease may be congenital in the context of a syndrome in cases involving children. Congenital lymphatic abnormalities, such as primary lymphatic hypoplasia or hyperplasia, are more common in the pediatric population. Most of pediatric chylous ascites cases (45%-70%) are congenital or idiopathic lymphatic abnormalities. Yellow-nail syndrome is a disorder of unclear etiology observed in childhood. It is a combination of yellow discolored nails, lymphedema, pleural effusions, and/or chylous ascites; these patients have hypoplastic lymphatics leading to chylous effusions. Battered child syndrome accounts for approximately 10% of chylous ascites cases in the pediatric population^[4]^. Any obstruction or damage to the lymphatic channels can result in chylous ascites. Chylous ascites is also a rare complication following AAA repair. And AAA repair is thus considered to be the most frequent cause of postoperative chylous ascites, accounting for approximately 80% of such cases^[2]^.

Abdominal distention is the most common presenting symptom. Pabst et al.^[3]^ reported that this disorder should be considered whenever persistent abdominal distention appears after aortic surgery. However, there is no radiological evidence to distinguish chylous ascites from other ascites-causing agents. CT is an important diagnostic tool in such cases, although it has low specificity. In case of strong clinical suspicion, CT is a useful diagnostic tool. First, it confirms the presence of ascites. It also reveals extra- and intraperitoneal collection of fluid. Chyle can be distinguished from blood on CT because of the difference in density; chyle has a low attenuation on CT. On the other hand, its density has the appearance of water, rendering it indistinguishable from other biological fluids, such as bile or simple ascites^[5]^. If the fluid drained appears milky when paracentesis is performed, the presence of chylous ascites can be suspected. The diagnosis can be made when the ratio of cholesterol to triglycerides in the paracentesis fluid is <1 and the triglyceride level is >110 mg/dL. However, the presence of chylomicrons in the lipoprotein analysis confirms the diagnosis^[6,7]^.

**Fig. 4:**
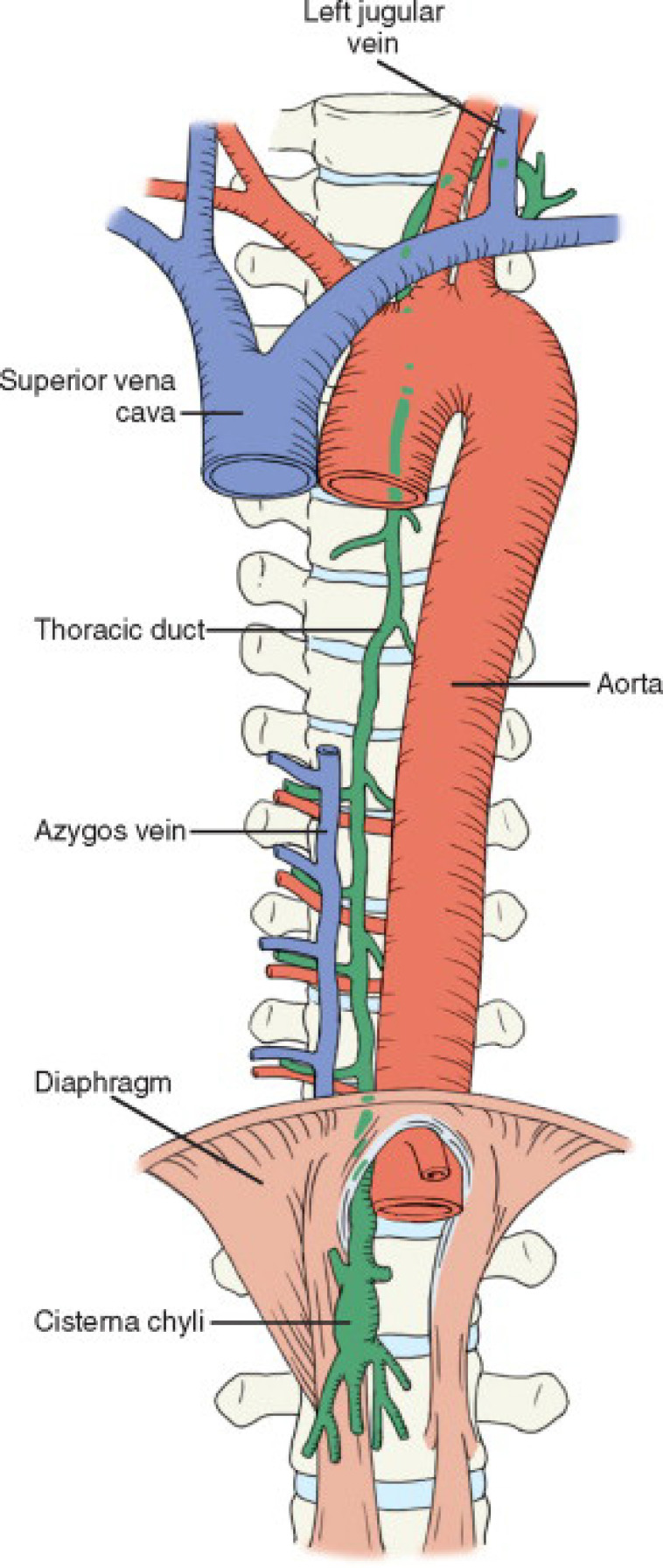
Cisterna chyli.

Lymph flows from the lymphatic vessels into the lymphatic trunks and then into the thoracic duct. The thoracic duct is the largest lymphatic channel in the body and it originates from the cisterna chyli located anterior to the bodies of the first and second lumbar vertebrae (Figure 4)^[8]^. About half of all individuals lack a true cisterna chyli. Instead of this anatomic structure, there is a confluence of lymphatics from which the thoracic duct originates^[9]^. Postoperative chylous ascites is commonly believed to occur as a result of direct trauma to the cisterna chyli or one of its branches. Nearly 50% of patients undergoing high aortic dissection present with lymph leakage^[2]^.

Lymph is a fluid containing proteins, immunoglobulins, lymphocytes, and products of the digestive process, such as lipid. Chylous leakage is accompanied by the loss of lipids, proteins, and electrolytes, resulting in nutritional defects and disturbance of the electrolytic equilibrium. In case of chylous leakage, significant metabolic and nutritional defects develop due to severe protein and vitamin loss. Persistent chylous leakage causes protein, fat soluble vitamins, lymphatic system cells, and antibody losses, which may result in immunodeficiency, coagulopathy, malnutrition, sepsis, and even death.

Treatments for chylous leakage depend on the severity of the injury. The most important step in the treatment of chylous ascites is the regulation of nutrition and liquid electrolyte balance. Conservative treatment, including paracentesis, fasting, diet containing medium-chain triglyceride, and total parenteral nutrition, is used as the first option for the treatment of chylous ascites. A diet with medium-chain triglyceride decreases the drainage of chyle into the lymphatics. The lymphatic vessels of the intestine have somatostatin receptors. Somatostatin and octreotide (a synthetic analog of somatostatin) have been successfully used in the treatment of postoperative chylothorax, particularly in neonatal and infantile chylothorax, in the recent years^[10,11]^. Some cases require surgical interventions, such as direct ligation of the chyle leak. However, our patient was successfully treated with total parenteral nutrition and somatostatin. Sarazin et al.^[12]^ recommended peritoneovenous shunt in the treatment of chylous ascites developing after ruptured AAA in patients who are at high risk for re-laparotomy complication. Barakat et al.^[13]^ attempted conservative treatment and peritoneovenous shunt for the treatment of chylous ascites developing after elective AAA repair, but they were not successful. Thus, they applied a ligature in the chylous leakage site through laparotomy. Di Luozzo et al.^[14]^ treated chylous ascites with extracorporeal peritoneovenous shunt in a 38-year-old patient with Marfan syndrome. Ohki et al.^[15]^ treated a patient who developed chylous ascites after AAA repair with total parenteral hyper-alimentation for 10 days following a low-fat diet. Perez et al.^[16]^ treated a patient who developed chylous ascites after AAA surgery with conservative and surgical treatment. But, because the patient did not respond to the treatment, they treated the patient with five cycles of retroperitoneal radiotherapy. Liu et al.^[17]^ reported a case of a refractory chylous ascites after pelvic lymphadenectomy, wherein they successfully performed laparoscopic ligation of the leaking lymphatics.

In our case, the chylous ascites evacuation intervention was performed immediately and the conservative treatment was initiated promptly after the diagnosis of chylous ascites. Oral intake was completely stopped, and total parenteral nutrition and somatostatin treatment were performed.

Chylous ascites developing after open surgical repair of AAA is a rare, but serious condition. A diagnosis of chylous ascites involves a history of operation, abdominal distention, and the analysis of ascites. Treatment modalities include serial paracentesis, a medium-chain triglyceride diet, total parenteral nutrition, and somatostatin as conservative management. Patients who do not respond to conservative treatment can be considered as candidates for peritoneovenous shunting and surgery. In the first treatment protocol of chylous ascites, we recommend drainage of the fluid in the abdominal cavity, followed by the cessation of oral intake and administration of total parenteral nutrition and somatostatin therapy.

**Table:** 

Author's roles & responsibilities	
HK	Substantial contributions to the conception or design of the work; or the acquisition, analysis, or interpretation of data for the work; drafting the work or revising it critically for important intellectual content; final approval of the version to be published
